# Electroshock Weapon Measurements: Instrumentation Requirements and Limitations

**Published:** 2017-04-12

**Authors:** N Paulter, D Jenkins, N Ichikawa

**Affiliations:** 1 National Institute of Standards and Technology, MD 20899, USA; 2 State College, The Pennsylvania State University, PA 16804-0030, USA; 3 Kogakuin University, Shinjuku, Tokyo 163-8677, Japan

**Keywords:** Electroshock weapons, Transient signals, Electrical pulses

## Abstract

Electroshock Weapons (ESWs) are a commonly used tool in the escalation of force arsenal for law enforcement and the military around the world. The ESWs provides a high-voltage low- current electrical shock (a pulse burst) that can temporarily incapacitate its target (typically a human). This shock is usually of sufficient energy to cause the individual to become temporarily incapacitated for up to a few seconds after the discharge is completed. It is important to accurately know the output of the ESW because of the serious safety ramifications if the ESW fails to operate properly. However, these transient ESW pulse outputs may have frequency content exceeding 100 MHz while simultaneously have durations greater than 10 s, and the impedance of the target may vary amongst targets and may vary between pulses of a given pulse burst for a given target. These facts greatly increase the challenges in performing high-fidelity reproducible measurements of the ESW transient signals. To ensure that the ESW operates properly requires special measurement instruments because of the bandwidth, duration, and amplitude of the output signals. Moreover, accurate measurement capability supports modelling and subsequent understanding of the physiological effects of ESW exposure.

## Introduction

**Figure 1:**
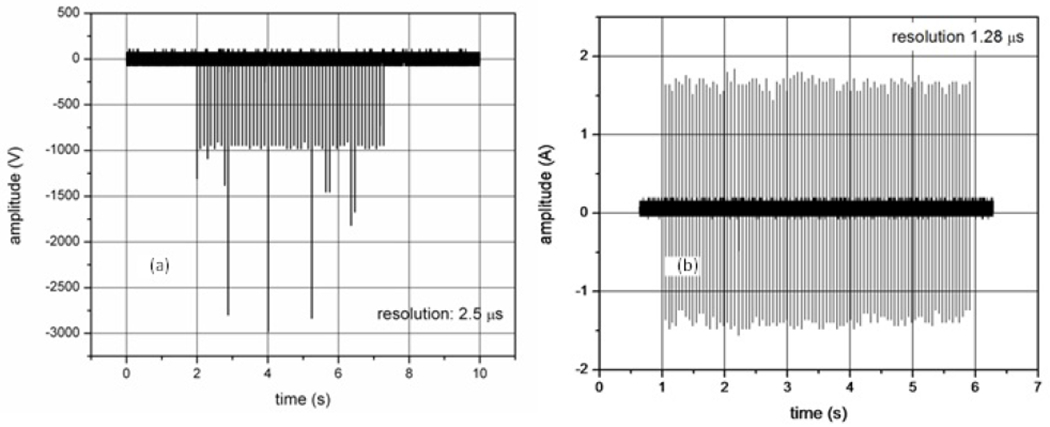
Pulse train from different ESW models. Plot (a) show the high-voltage output with the sampling resolution or interval set to 2.5 μs. Plot (b) shows the current output with a sampling resolution of 1.28 μs. The variation of the peak amplitude in (a) is caused by a nonopotimal sampling interval (or resolution).

**Figure 2:**
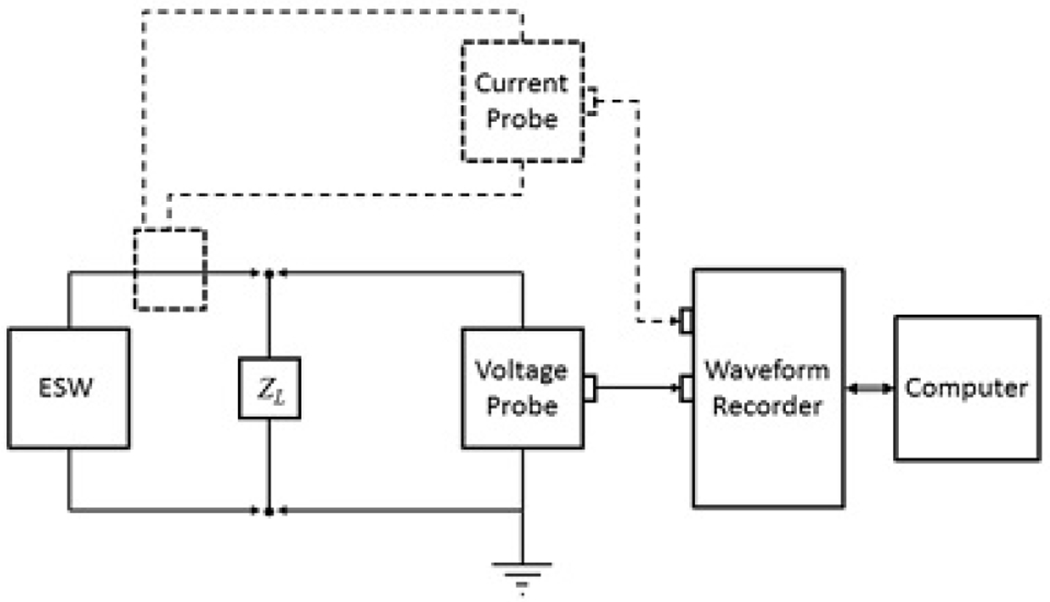
ESW measurement system.

The electrical output of the ESW is a pulse train burst (Figures 1 and 2) containing many tens to hundreds of nominally identical electrical pulses with a total pulse burst duration ranging from less than 1 s to greater than 10 s. The pulses in a burst may have fast transitions ranging from approximately a couple of nanoseconds to a more than a few microseconds in duration [1–3]. The pulses may be bipolar and may exhibit aberrations, where these aberrations are dependent on the electrical load attached to the ESW.

Consequently, waveform parameters should be extracted from the pulse train that accurately and reproducibly describe the output of the ESW. Current commercially-available off-the-shelf instruments cannot provide the necessary measurement capability to capture the detail and duration of the ESW current or high-voltage outputs. We describe how measurement system shortfalls influence waveform fidelity and provide guidance on minimally-acceptable system performance requirements. Although we have developed a measurement system to provide the requisite metrological capability, we hope that the information presented here would elicit interest in the development of capable measurement systems suitable for use by metrology and calibration labs to support medical research labs and ESW technology developers [4].

## Measurement system general considerations

A general diagram for an ESW measurement system is shown in Figure 2. Tables 1 and 2 contain performance specifications for the expected components of the measurement system. These specifications were determined to be applicable for metrology-quality measurements of the output of ESW. Figure 2 shows the testing of an ESW being tested, where the dotted lines show a measurement configuration for measuring the current output of the ESW.

## Test Methods

To measure the electrical output of an ESW, it must be connected to an appropriate electrical load. This electrical load should emulate that expected during normal used of the ESW and, in a measurement, is terminally connected to the ESW. ESW testing requires measuring both the current and High-Voltage (HV) output, thus one current transducer and one HV transducer are required.

The HV transducer typically has high input-impedance and is attached in parallel to the load resistor. This transducer provides electrical isolation between the HV ESW output and the low-voltage input of the waveform recorder. The current transducer provides similar electrical isolation. The current passing through the electrical load is sensed by the transducer and provides a proportional low-voltage output.

Testing of the ESW requires that the ESW be terminated into electrical loads that emulate the electrical load presented by the expected target, where the output of a given ESW may be a function of the electrical load [4]. To emulate the typical electrical load of targets, materials such as solid polymeric (carbon-black loaded fluoropolymer) materials, which has the same nominal bulk electrical conductivity of human muscle tissue (about 0.8 S/m from about 10 Hz to 100 MHz), and saline solutions (electrical conductivity of about 0.8 S/m), have been examined [5][7].

**Figure 3:**
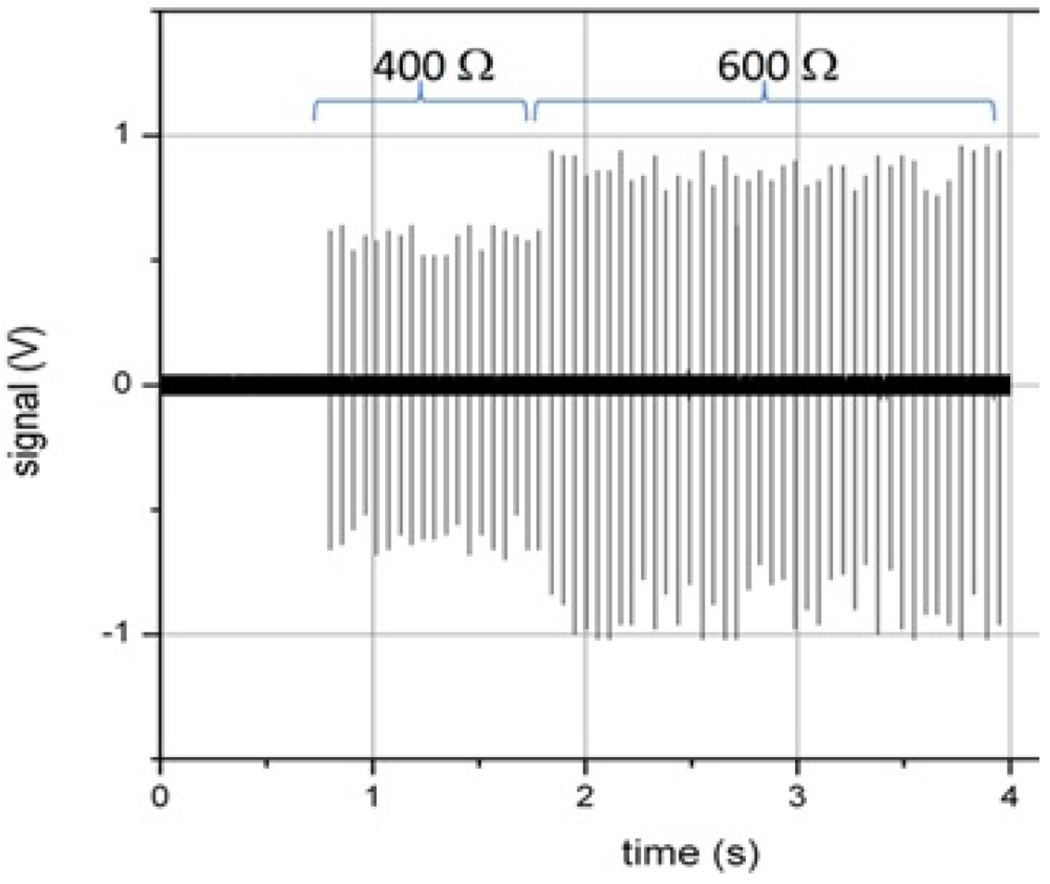
Output from an ESW showing an abrupt change in load between the 19th and 20th pulses in the pulse train.

However, greater measurement reproducibility and ease of making electrical connection is obtained using high-voltage-rated resistors than by using the phantom or saline solution. Some ESW manufacturers claim that their ESW models can sense changes in the electrical load presented by the target during an exposure and then adapt the output of the pulses to deliver a constant energy to the target. The effect of high voltage exposure on impedance change has also been observed experimentally [8]. Figure 3 shows the output current of an ESW while abruptly changing the load from 400 Ω to 600 Ω between the 19th and 20th pulses. This particular model ESW does not have the ability to adjust its output in real time.

## Parameters Measured

The waveform parameters of peak amplitudes of voltage and/or current, total charge (net, positive, or negative), partial charge (charge contained within defined parts of the waveform), pulse duration, pulse repetition rate, number of pulses, and pulse burst duration are the most commonly cited parameters for describing ESW performance. These parameters are all affected by noise, sampling rate, and the attenuation bandwidth of the measurement system (Sec. II.D). The temporal parameters (durations and rates) and charge parameters will also be dependent on user-defined values for amplitude reference levels, and initial and final summation instants (Sec. II.E.).

The terms listed below are used to describe the ESW electrical output and are defined in the International Electrotechnical Commission (IEC) 62792 [9], the IEC 62754 [10] (equivalently the IEEE Std. 181 [11]). Note, the measurements of the ESW output are single events (single shot) and averaging between successive events may not be possible or advisable because of signal repeatability.

Pulse duration, TP, is computed using [10][11]:

(1) $$T_{p}=\left|t_{2,x\%}-t_{1,x\%}\right|$$

where t2,x% is the reference level instant for the first transition of the pulse and ${\text{t}}_{1,\text{x}\%}$ is the reference level instant for the second transition of the pulse. Similarly pulse separation, pulse period, etc., are a function of t2,x% and t1,x%. The TP is a function of user-defined reference levels, sampling rate, noise, and measurement system attenuation bandwidth.

Another important parameter for ESW characterization is the charge delivered to a load. This charge is computed using either the average over the entire current waveform (for net charge) or the average over user-specified parts of the current waveform (for positive or negative charge). The average value of a pulse burst is computed using [9]:

(2) $${\bar{y}}_{i}=\left(\frac{1}{M_{i}}\right)\sum_{j=1}^{M_{i}}y_{j,}i=1,......N,$$

where y is charge, $M_{i}$ is the number of waveform samples in the ith waveform sub-epochs, N is the number of samples (elements) in a waveform sub-epoch, i is the index for the waveform sub- epoch (one sub-epoch for each pulse in a pulse burst), and j is the summation index. One of the pulses in the pulse train correlates to a waveform sub-epoch. For simplicity, consider only one waveform sub-epoch, so (2) reduces to:

(3) $$\bar{y}=\left(\frac{1}{M}\right)\sum_{j=1}^{M}y_{j}$$

Net charge, $Q_{net}$ , is then computed from (3) using [9]:

(4) $$Q_{net}=\bar{y}T$$

Where T is the duration of the waveform epoch or the summation interval defined by the user. Measurement and computation processes and variables that affect yi and T will influence the value of Qnet. Therefore, user defined epochs will affect $Q_{net}$ . Furthermore, ESW industry practice is to set amplitude thresholds that exclude yi from the summation, and this will affect $Q_{net}$ . The $Q_{net}$ is a function of user-defined reference levels and threshold values, system attenuation bandwidth, noise, and the sampling rate.

## Instrument Effects

**Figure 4:**
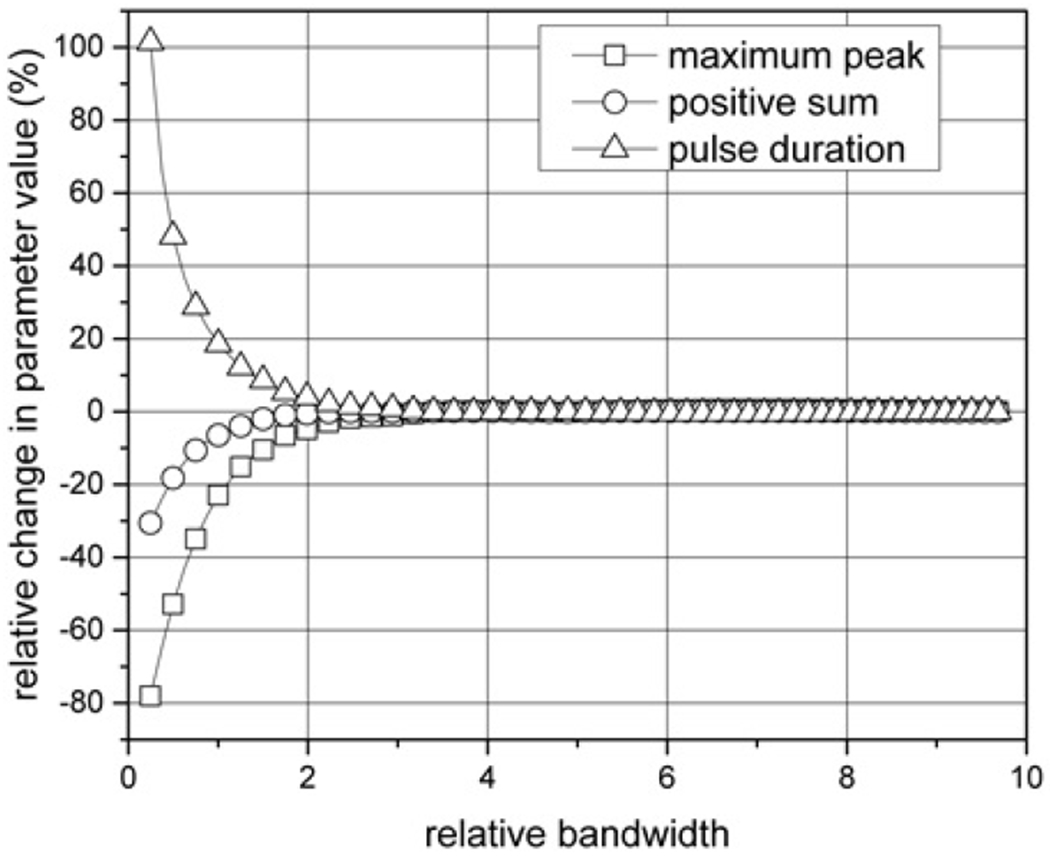
Relative bandwidth and its effect on maximum peak, positive sum, and pulse duration. The reference parameter values are taken at a relative bandwidth of 10, which is the highest bandwidth.

The effects of the ESW measurement system are computed using numerical simulations and, therefore, the waveform values and axis labels in Figure 4 are unit less. The measurement system’s 3 dB attenuation bandwidth affects the maximum peak; positive, negative, and total sums; and pulse duration of the ESW output waveforms. The effect of attenuation bandwidth on some of these parameters is shown in Figure 4. The relative bandwidth, ${BW}_{R}$ , of the ESW measurement system is computed using:

(5) $${BW}_{R}=\frac{{BW}_{sig}}{{BW}_{sys}}$$

where $BW_{sig}$ and $BW_{sys}$ are the −3 dB attenuation bandwidths of the ESW output signal and of the measurement system. For the current waveform, this sum will be related to the charge delivered to the load per Equation (4).

**Figure 5:**
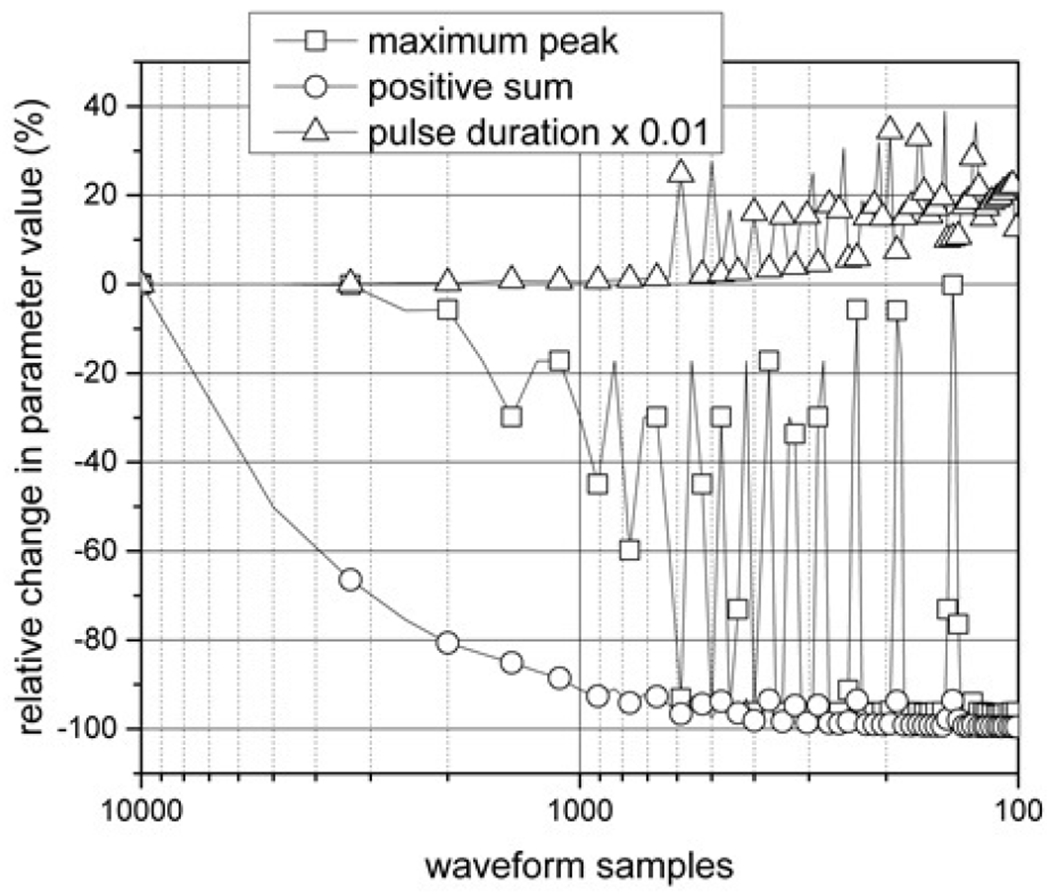
The effect of waveform samples on ESW relative performance parameters of maximum peak, positive sum, and pulse duration are shown. The relative pulse duration values were multiplied by 0.01 to plot on the same scale as the other relative performance parameters. The reference parameter values are taken at 10,000 waveform samples, which is the highest sampling rate.

The sampling rate used in the ESW measurement can affect the ESW output waveforms. Specifically, detail of the ESW output may not be captured if the sampling rate is to low, and, consequently, any parameters depending on accurate temporal information may be lost. Figure 5 shows the effect of sampling rate, or equivalently the number of waveform samples, on pulse duration, peak amplitude, and the sum of the positive waveform values. Post-sampling filtering was not applied to prevent bandwidth reduction.

ESW measured values of peak amplitude and pulse durations are also sensitive to instrument and signal noise. Since these are single shot measurements, signal averaging cannot be used to reduce the effect of noise. Noise causes erroneous waveform values of peak amplitude, reference levels, and waveform instants, all of which are used in the computation of the parameters previously described.

## User-defined Parameter Effects

**Figure 6:**
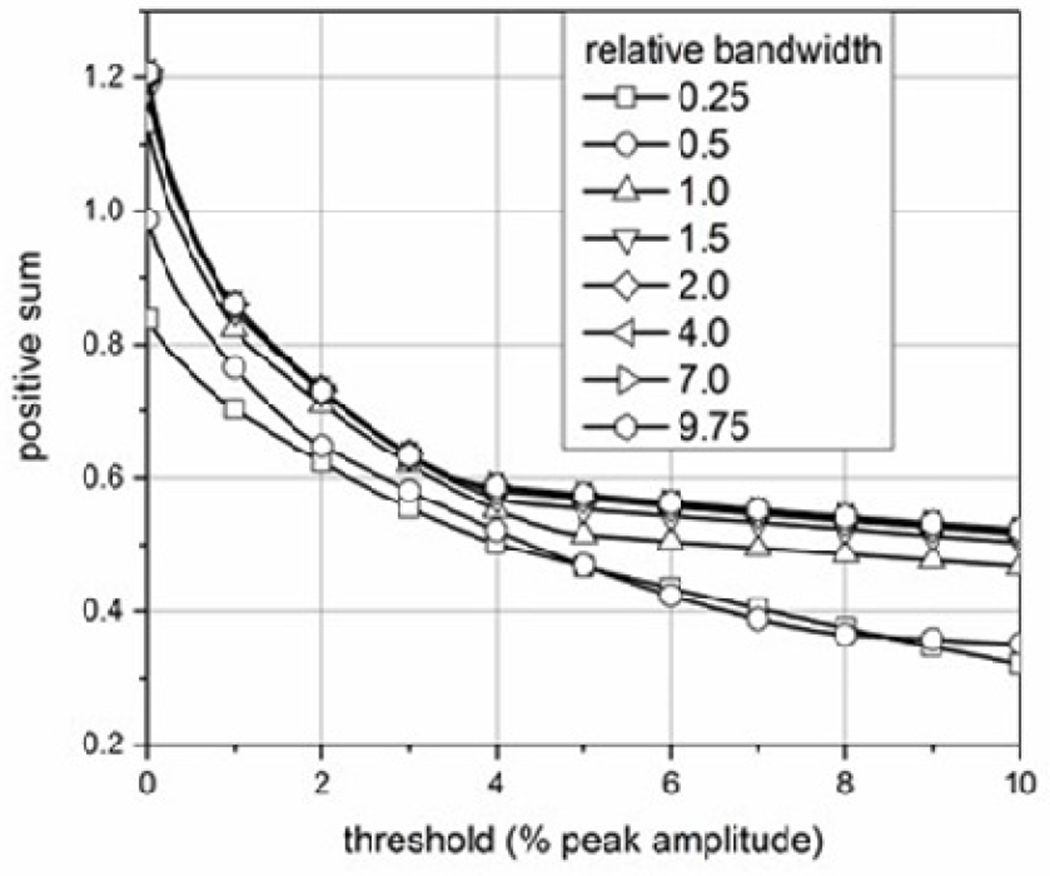
The effect of increasing summation threshold on the positive sum for waveforms with different relative bandwidths.

The user of the ESW measurement system defines the percent reference levels and reference instants (as described in the IEC 62792) that are used in the computation of the ESW waveform parameters, and these user-defined values will affect the value of those ESW waveform parameters. As an example, Figure 6 shows that increasing the reference level (or amplitude summation threshold) decreases the sum of the positive waveform values. This summation threshold is given as percentage of the peak amplitude. The change in the positive sum is the greatest for small increases in the summation threshold and is more pronounced for the waveforms with higher relative bandwidth. Different waveform profiles will demonstrate different sensitivities to changes in the summation threshold. The effects of reference instants, which would define the interval for summation, has similar effects to that shown here.

## Summary

Electroshock weapons provide a unique measurement challenge to accurately capture its output, which is a single burst of high-bandwidth high-voltage pulses that often exceeds a few seconds. This uniqueness constrains the options for the measurement system required to capture the waveforms with sufficient fidelity. Inadequate measurement systems and poorly-defined user references can significantly reduce the accuracy of the measured waveform parameter.

## Figures and Tables

**Table 1:** Waveform recorder minimum performance specifications.

Parameter	Values
Analog bandwidth (MHz)	≥500
Sampling rate (Samples/s)	≥1 × 10^9^
Epoch, minimum (s)	≥10
Signal-to-noise ratio (SNR) (dB) [IEEE 1057, Clause 8.3]	≥40
Signal-to-noise-and-distortion ratio (SINAD) (dB) [IEEE 1057, Clause 8.2]	≥40
Spurious-free dynamic range (SFDR) (dB) [IEEE 1057, Clause 8.8]	≥50
Effective number of bits (ENOB) (bits) [IEEE 1057, Clause 8.5]	≥7
Input impedance: matched to probe impedance, *Z_probe_*	*Z_probe_* ± 0.02 *Z_probe_*
Input impedance: not matched to *Z_probe_*	≥10 *Z_probe_*

**Table 2:** Connector, cable, load, and probe minimum performance specifications.

Parameter	Values
Impedance: Cable, connector	50 Ω ± 2 Ω
Impedance, output: High-voltage probe	*Z_WR_* ± 0.05 *Z_WR_* : To match to a waveform recorder with input impedance, *Z_WR_* , of approximately 50 Ω <0.1 *Z_WR_* for *Z_WR_* ≠ 50Ω
Impedance, output: Current probe	*Z_WR_* ± 0.05 *Z_WR_* for matching to a waveform recorder with a nominal input impedance, *Z_WR_* , of 50 Ω <0.1 *Z_WR_* , for *Z_WR_* ≠ 50Ω
Analog bandwidth: Cables, connectors	>1 GHz
Resistance: Electrical load	400 Ω ± 4Ω 600 Ω ± 6 Ω 1000 Ω ± 10Ω
Inductance: Electrical load	<0.01 *L_ESW_* or 20 nH, whichever is greater, where *L_ESW_* is the self-inductance of the wire connecting the barbs and body of an ESW
Analog bandwidth: High-voltage probe	≥100 MHz
Analog bandwidth: Current probe	≥200 MHz
Current-voltage ratio: Current probe	Appropriate for ESW output and *Z_WR_*
Hi-voltage-voltage ratio: High-voltage probe	Appropriate for ESW output and *Z_WR_*
